# Gadolinium-Coated Mesoporous Silica Nanoparticle for Magnetic Resonance Imaging

**DOI:** 10.3389/fchem.2022.837032

**Published:** 2022-02-15

**Authors:** Zhongtao Li, Jing Guo, Mengmeng Zhang, Guohua Li, Liguo Hao

**Affiliations:** ^1^ Department of Molecular Imaging, School of Medical Technology, Qiqihar Medical University, Qiqihar, China; ^2^ Department of Radiology, The First Affiliated Hospital of Qiqihar Medical University, Qiqihar, China; ^3^ Department of Molecular Imaging, The First Affiliated Hospital of Qiqihar Medical University, Qiqihar, China

**Keywords:** gadolinium oxide, mesoporous silica nanoparticle, magnetic resonance imaging, cytotoxicity, contrast agent

## Abstract

Magnetic resonance molecular imaging can provide anatomic, functional and molecular information. However, because of the intrinsically low sensitivity of magnetic resonance imaging (MRI), high-performance MRI contrast agents are required to generate powerful image information for image diagnosis. Herein, we describe a novel *T*_*1*_ contrast agent with magnetic-imaging properties facilitated by the gadolinium oxide (Gd_2_O_3_) doping of mesoporous silica nanoparticles (MSN). The size, morphology, composition, MRI relaxivity (*r*_*1*_), surface area and pore size of these nanoparticles were evaluated following their conjugation with Gd_2_O_3_ to produce Gd_2_O_3_@MSN. This unique structure led to a significant enhancement in *T*_*1*_ contrast with longitudinal relaxivity (*r*_*1*_) as high as 51.85 ± 1.38 mM^−1^s^−1^. Gd_2_O_3_@MSN has a larger *T*_*1*_ relaxivity than commercial gadolinium diethylene triamine pentaacetate (Gd-DTPA), likely due to the geometrical confinement effect of silica nanoparticles. These results suggest that we could successfully prepare a novel high-performance *T*_*1*_ contrast agent, which may be a potential candidate for *in-vivo* MRI.

## 1 Introduction

Magnetic resonance imaging (MRI) is non-invasive and produces high-resolution morphological and innate three-dimensional image resolution without the risk of radiation damage, making it a critical clinical diagnostic tool. MRI contrast agents are a specific substrate used in these technologies to help improve the contrast between normal and abnormal tissues. Gadolinium diethylene triamine pentaacetate (Gd-DTPA) remains the most common MRI contrast agent as it can enhance the brightness of the region of interest (ROI; positive contrast) (Shin et al., 2015; Ni et al., 2017; Shen et al., 2017). However,Gd-DTPA is a small-molecule contrast agent and suffers from relatively low sensitivity, specificity, and relaxivity, which limits its applications in more complex diagnostic settings (Fraum et al., 2017; Vikas et al., 2017). This implies that alternative contrast agents, specifically those commonly referred to as gadolinium-based *T*_*1*_ contrast materials have begun to attract more attention.

In addition, nano-drug delivery systems (NDDS) are currently amongst the most evaluated drug delivery systems in the world. These NDDS combine a therapeutic payload with nano-sized carriers (such as liposomes, gold nanoparticles, polymeric micelles, and mesoporous silica) to reduce side effects and prolong circulation time (Mikada et al., 2017; Layek et al., 2020). Thus, the introduction of MRI contrast agents into an NDDS may both extend blood circulation time and improve MRI performance (because of the shortened longitudinal proton relaxation time of the surrounding water molecules for *T*_*1*_-type contrast agents) (Lin et al., 2004; Parvesh et al., 2012; Li et al., 2014; Hu et al., 2015; Deng et al., 2016; Wang and Sun, 2020). Of the common NDDS carriers mesoporous silica nanoparticles (MSNs) are widely used in the carriers for drug delivery and biosensing and are likely to be the most appropriate carrier for gadolinium owing to their high surface area, ease of preparation, good biocompatibility, and mesoporous structure (Lin et al., 2004; Taylor et al., 2008; Xia et al., 2019). Additionally, the overall structure is composed of silica and Si-OH groups. Si-O-Si frameworks are quitestable and silica degradation is relatively difficult under physiological conditions, which means that these particles are likely to facilitate good loading of Gd_2_O_3_ but inhibit the release of free Gd^3+^ reducing its toxic effects.

**SCHEME 1:**
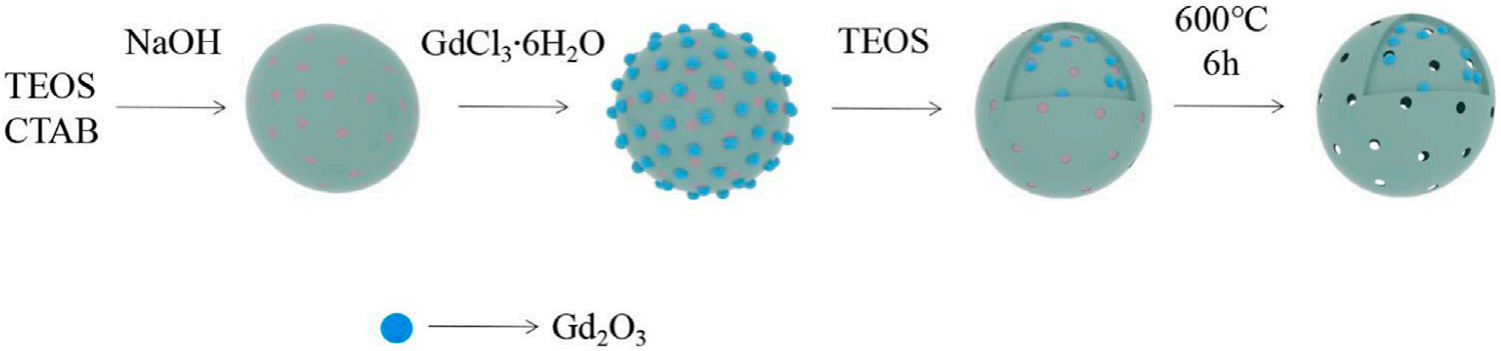
Illustration of the preparation of Gd^3+^-incorporated mesoporous silica nanoparticles (MSN) (Gd_2_O_3_@MSN) particles for magnetic resonance imaging.

Herein, we report a novel synthesis system for producing Gd^3+^-incorporated MSN (Gd_2_O_3_@MSN), which are characterised by a mesoporous structure, higher surface area and high *T*_*1*_ relaxivity. These nanoparticles (NPs) are easy to prepare and modify, present with low-cost, and possess desirable MRI contrast-enhancement properties, thus making them suitable for the creation of more specific, and possibly even targeted, contrast agents for molecular MRI and could help provide real-time feedback for treatment outcomes (Scheme 1), potentially enhancing the clinical utility of MRI.

## 2 Materials and Methods

### 2.1 Materials

Hexadecyl trimethyl ammonium bromide (CTAB, 99%) was purchased from Coolaber Science & Technology. Tetraethoxysilane (TEOS, 99%) was purchased from Fuchen Chemical Reagents (Tianjin, China). 3-Aminopropyl-triethoxysilane (99%) was purchased from Macklin Biochemical Co., Ltd. (Shanghai, China). Gadolinium (III) chloride hexahydrate (GdCl_3_·6H_2_O, 99.9%) was purchased from Aladdin Biochemical Technology Co., Ltd. (Shanghai China).

### 2.2 Preparation of Gd_2_O_3_@MSN

Briefly, NaOH (140 mg) and CTAB (500 mg) were dissolved in 220 ml of deionised water and stirred (300 rpm) at 80°C for 1 h. Next, we slowly added TEOS (1.8 ml) in a dropwise manner to this suspension while maintaining the stirring (250 rpm) of the recipient solution. This mixture was then stirred for another 2 h and then 20 ml of GdCl_3_·6H_2_O aqueous solution (5 mg/ml) was quickly added into the mixture. One hour later, an additional 0.7 ml of TEOS was added to these samples, left for an additional 2 h, centrifuged, washed with ethanol and deionised water, and then dried in an oven at 50°C for 24 h. The resulting Gd_2_O_3_@MSN were collected and calcined at 600°C for 6 h to remove CTAB surfactants.

### 2.3 Characterizations

Gd_2_O_3_@MSN (1 mg/ml) particle size and zeta potential was confirmed by dynamic light scattering (Malvern Zetasizer Nano ZS system, Malvern, Worcestershire, United Kingdom). We then performed transmission electron microscopy (TEM) (FEI Talos F200S, United States) to examine the surface morphology of the Gd_2_O_3_@MSN particles. Their composition was evaluated by energy dispersive X-ray spectroscopy (EDS). Scanning transmission electron microscopy high-angle annular dark-field (STEM-HAADF) images and energy-dispersive X-ray (EDX) element mapping images were obtained using an FEI Talos F200S microscope at an accelerating voltage of 300 kV. The analysis of the N_2-_adsorption isotherms was performed using Barrett–Joyner–Halenda (BJH) analysis (Micromeritics ASAP-2460, Norcross, GA, United States). The surface area, total pore volume, and average pore distribution curves for the MSNs were determined using the Brunauer–Emmett–Teller (BET) method. Fourier transform infrared refraction (FT-IR, RF-5301PC, Shimadzu, Japan) analyses of the Gd_2_O_3_@MSN particles were performed in the range of 400–4,000 cm^−1^ or structural characterization.

### 2.4 *T*_*1*_ Relaxivity and *in-vitro* Magnetic Resonance Imaging of as-prepared NPs

The Gd^3+^ concentration of the doped MSNs was proved using Inductively-coupled plasma mass spectrometry (ICP-MS, Agilent 720 ES, United States), and ICP-MS was also used to detect whether free Gd elements were dissociated from Gd_2_O_3_@MSNs when immersed in phosphate-buffered saline (PBS) at different pH (7.4, 5.5 and 4.5) for 48 h. Next we evaluated the *T*_*1*_ relaxivity of Gd_2_O_3_@MSN and Gd-DTPA with different molar concentrations of gadolinium when kept in 1% agarose solution using a 0.5 T NMI20 Analyst NMR system (Niumag Analytical Instrument Corporation, Sunzhou, China) (Repeat three times for each sample) set to apply the following parameters:SW:100 kHz, TW: 3,000 ms, RFD: 0.08 ms, NS: 8, TE: 1 ms and NTI: 25 (in pure water, 37°C), and the *T*_*1*_ graph was obtained using the inversion recovery (*T*_*1*_-TSE) sequence: TR/TI: 3,000/20 ms, TE: 20 ms, matrix: 256 × 192, layer thickness: 3 mm, FOV: 90 × 120 mm.

### 2.5 *In-Vitro* Evaluations

#### 2.5.1 Cell Culture

The human pancreatic cancer cell line AsPC-1, PaCa-2 and 4T1 breast cancer line were purchased from the Shanghai Cell Bank of the Chinese Academy of Sciences and cultured in Roswell Park Memorial Institute 1,640 (RPMI 1640) medium containing 10% (v/v) foetal bovine serum (FBS) and 1% penicillin/streptomycin. The cell lines were cultured using regular cell culture conditions (37°C with 5% CO_2_).

#### 2.5.2 Cytotoxicity Assay

AsPC-1, PaCa-2 and 4T1 cells were separately plated in 96-well plates (5 × 10^3^cells/well) with 100 μL of medium and incubated for 24 h before the culture medium was replaced with 100 μL of RPMI 1640 supplemented with different concentrations of Gd_2_O_3_@MSN (5, 25, 50, 100, 150 and 200 μg/ml) and incubated for an additional 24 h. The drug-containing culture medium was then removed and replaced with 100 μL of fresh medium and 10 μL of CCK-8 and incubated for 2 h before measuring the absorbance at 490 nm using a plate reader (SAFIRE2, TECAN, Switzerland). Cell viability was then calculated using the following equation: Cell viability (%) = (A_treated_–A_blank_)/(A_control_–A_blank_) × 100%. where A_treated_, A_control_ and A_blank_ represent the absorbance of the treated, control and blank wells, respectively.

### 2.6 *In-Vivo* Evaluations

#### 2.6.1 Experimental Animals

Male SPF-grade Sprague–Dawley (SD) rats (180 ± 10 g) were purchased from Liaoning Changsheng biotechnology Co., Ltd. (SXK2020-0001) and housed as prescribed. The animal experiments were approved by the Animal Ethics Committee of Qiqihar Medical University (No. QMU-AECC-2021-168).

#### 2.6.2 *In-Vivo* Toxicity Studies

Ten healthy SD rats (180 ± 10 g) were randomly divided into two groups (five rats in each). The rats were then injected with 100 mg/kg Gd_2_O_3_@MSN or saline. After 7 days, all the rats were sacrificed and approximately 3 ml of blood was collected from each rat for blood chemistry evaluations immediately before being euthanised. Then, the major organs, namely the heart, liver, spleen, lung, and kidneys, were harvested from those rats for H&E staining and histopathological examination (Leica-DM4B digital microscope, Germany).

#### 2.6.3 *In-Vivo* Magnetic Resonance Imaging Studies

These experiments were performed using a Philips (Achieva 3.0 T) MRI scanner with 8-channel carotid wall imaging special phased array coil. Male SD rats were selected for *T*_*1*_-weighted MRI from each group (*n* = 3) and injected with Gd_2_O_3_@MSN or Gd-DTPA at 0.5 mg of Gd^3+^ per kg of body weight. The images were then produced using a *T*_*1*_ sequence with the following parameters: TR/TE = 650/10 ms, thickness = 3 mm, 192 × 192 matrices, FOV = 130 × 130 mm and flip angle = 90°. The signal-to-noise ratio (SNR) for each image was then calculated by analysing each ROI (in each image). Contrast enhancement was defined as an increase in SNR after injection using the following equation:

ΔSNR=(SNRpost−SNRpre)/SNRpre

All image data were transferred to a remote computer for analysis.

### 2.7 Statistical Analysis

Nanoparticle size was analysed using the Nano Measure 1.2 software, and SPSS 20.0 (SPSS Inc., Chicago, United States) was used for data management and statistical analysis (Student’s t-test for unpaired data). The data are expressed as the mean ± standard deviation. A *p*-value of <0.05 is considered statistically significant and the SNR values were determined using ImageJ.

## 3 Results and Discussion

### 3.1 Preparation and Characterization of Gd_2_O_3_@MSN

**SCHEME 2:**
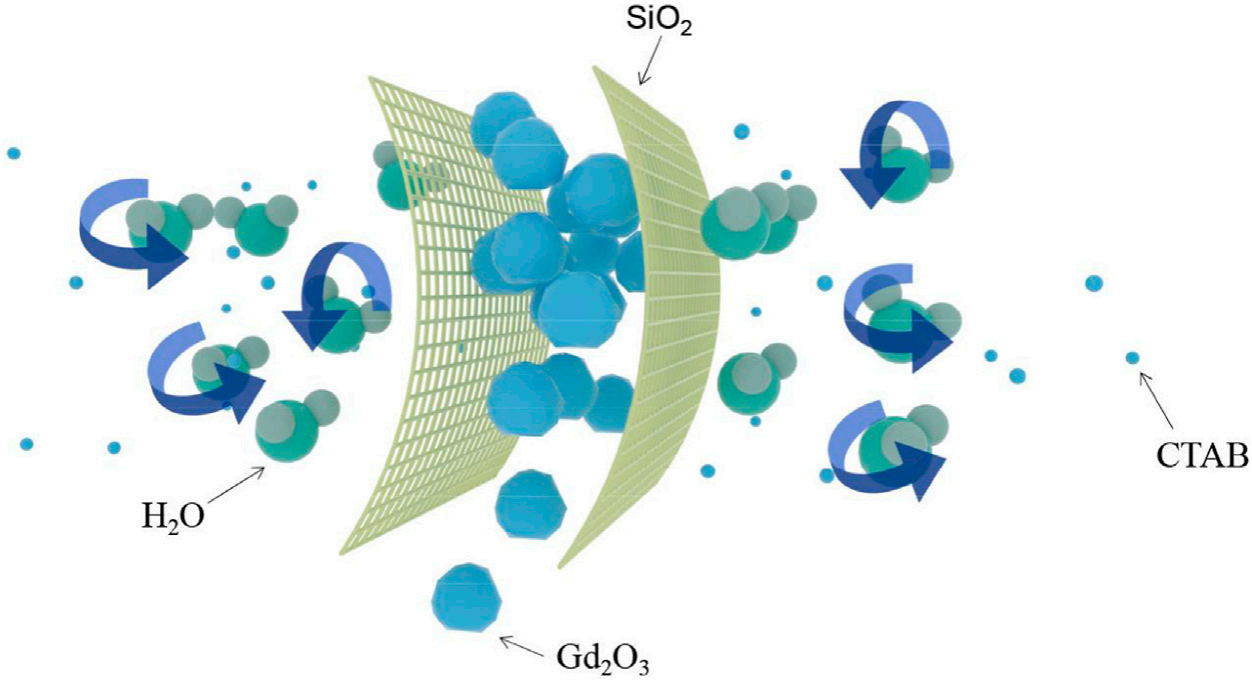
The removal of hexadecyl trimethyl ammonium bromide and the formation of Gd_2_O_3_.

Our preparation used CTAB as the template and TEOS as the main silica source. First, solid silica nanospheres containing the CTAB template were prepared and then Gd^3+^ was converted to Gd(OH)_3_ in alkaline solution (pH = 9), before being used to coat the surface of the SiO_2_ nanospheres. Once the *mesoporous* silica *shell* successfully coated the solid core, the final product was calcined at 600°C for 6 h to remove the CTAB template and dehydrate Gd(OH)_3_ to Gd_2_O_3_ (Scheme 2).

**FIGURE 1:**
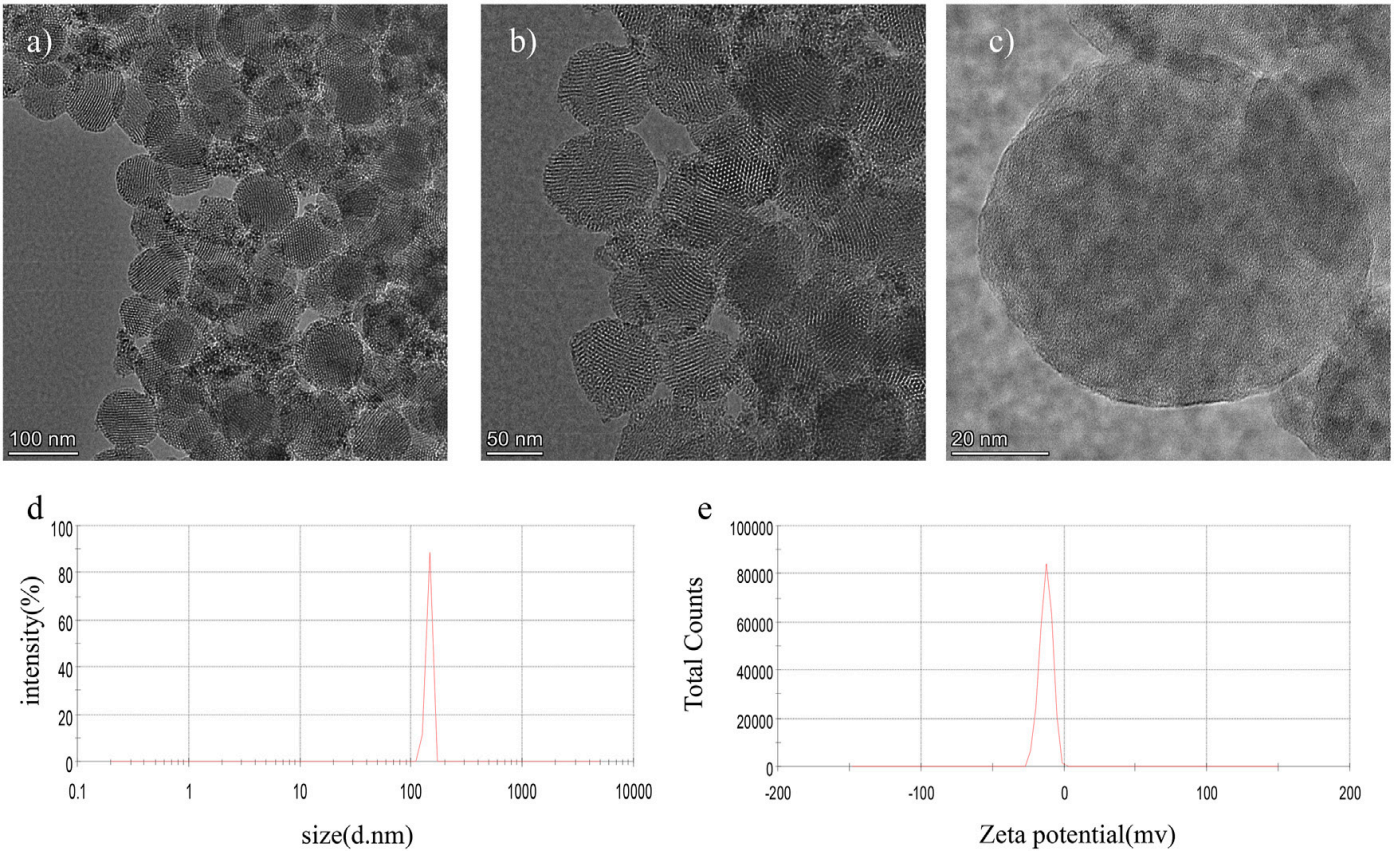
Structural evaluation of Gd_2_O_3_@mesoporous silica nanoparticles by **(A–C)** transmission electron microscopy; **(D)** dynamic light scattering; and **(E)** zeta potential evaluation.

Figure 1 described the structural characteristics of the prepared Gd_2_O_3_@MSN. TEM (Figures 1A–C) revealed that these NPs were spherical or ellipsoid in shape and of uniform size and distribution, and the mean diameter of these NPs was determined to be 86.85 ± 10.44 nm. Dynamic light scattering (DLS) curves (Figure 1D) revealed that the average diameter of the *NPs* was 162.50 nm, suggesting that the DLS measured these NPs as slightly larger than the TEM, which might be the result of the rehydration of the NPs in the aqueous solution used for the DLS evaluation. As expected, the zeta potential of the MSN was negative (Figure 1E), whereas the moderate size of these particles should help avoid renal clearance and uptake to the reticuloendothelial system (RES) in the liver, which were both essential for increasing circulation time (Phillps et al., 2010; Koo et al., 2011; Shao et al., 2011; O’Connell et al., 2017; Jiang et al., 2019).

**FIGURE 2:**
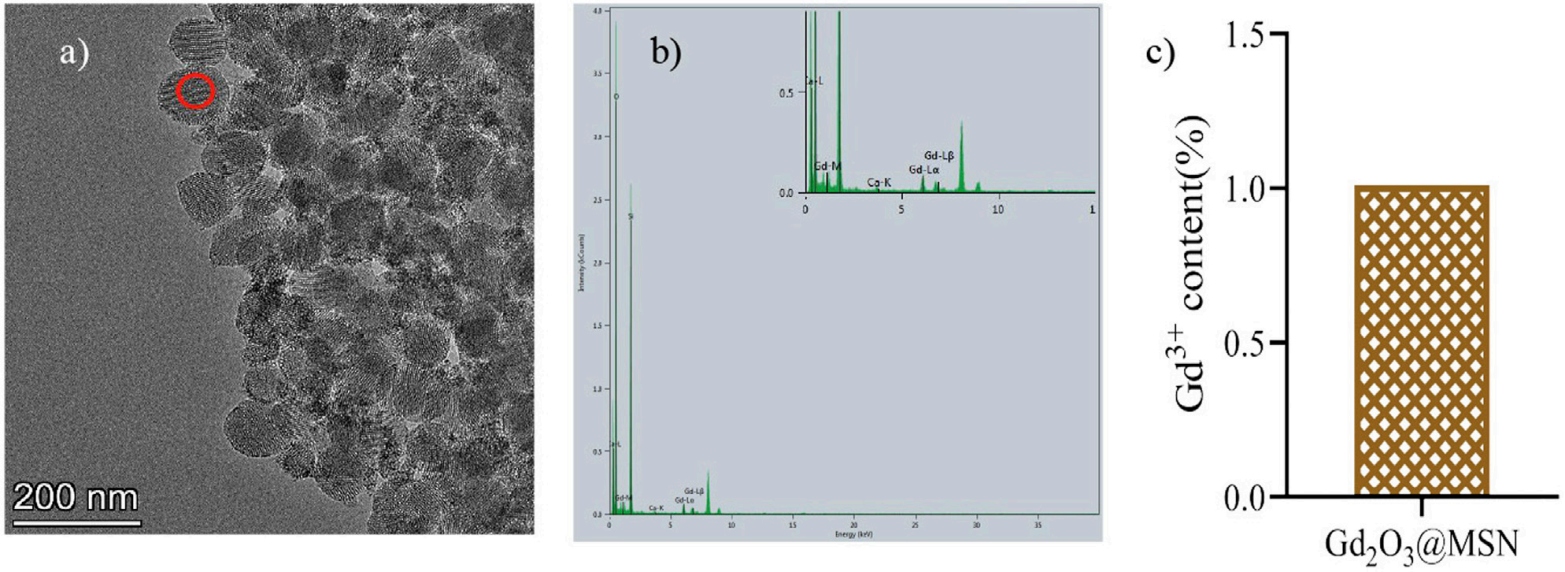
**(A)** Transmission electron microscopy images and **(B)** Energy dispersive spectrometer spectra of the Gd_2_O_3_@mesoporous silica nanoparticles (MSN) particles; **(C)** the Gd content in Gd_2_O_3_@MSN as determined by Inductively-coupled plasma mass spectrometry.

**FIGURE 3:**
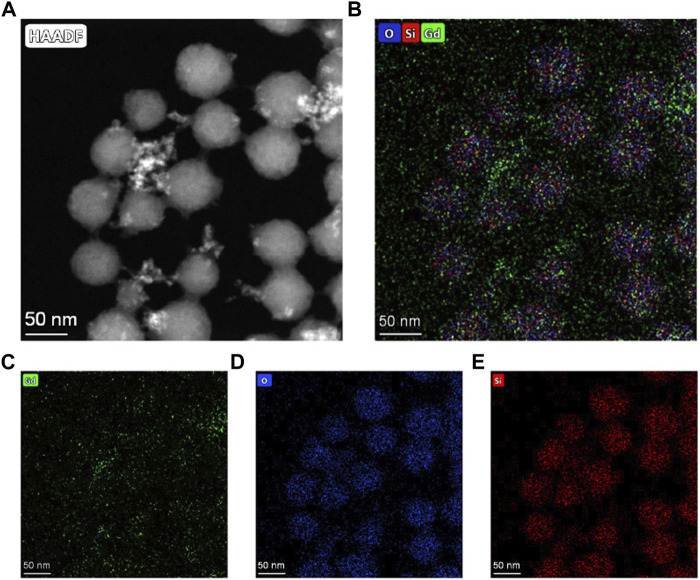
**(A)** Scanning transmission electron microscopy high-angle annular dark-field images of Gd_2_O_3_@MSN nanocomposites and **(B–E)** Elemental mapping images of Gd_2_O_3_@MSN nanocomposites, confirming that Gd^3+^ elements were evenly distributed across MSN structure.

The EDS (Figure 2B) spectrum confirmed that the red circle appearing in Figure 2A indicates Gd-existence with an atomic fraction of 0.91% (atom.%) Gd (Table 1). ICP-MS (Figure 2C) determined the overall Gd concentration to be 1.01 (atom. %). We then investigated the structure of NPs in detail. STEM-HAADF images (Figure 3A) and EDX elemental mapping images showed the significant and homogeneous signal of Gd in MSNs (Figures 3B–E), indicating the Gd^3+^ elements were evenly distributed across MSN structure.

**TABLE 1:** Results of energy dispersive X-ray spectroscopy analysis of the Gd2O3@mesoporous silica nanoparticles particles.

Location	Si (atom.%)	O (atom.%)	Gd (atom.%)
Red circle	30.53	68.56	0.91

**FIGURE 4:**
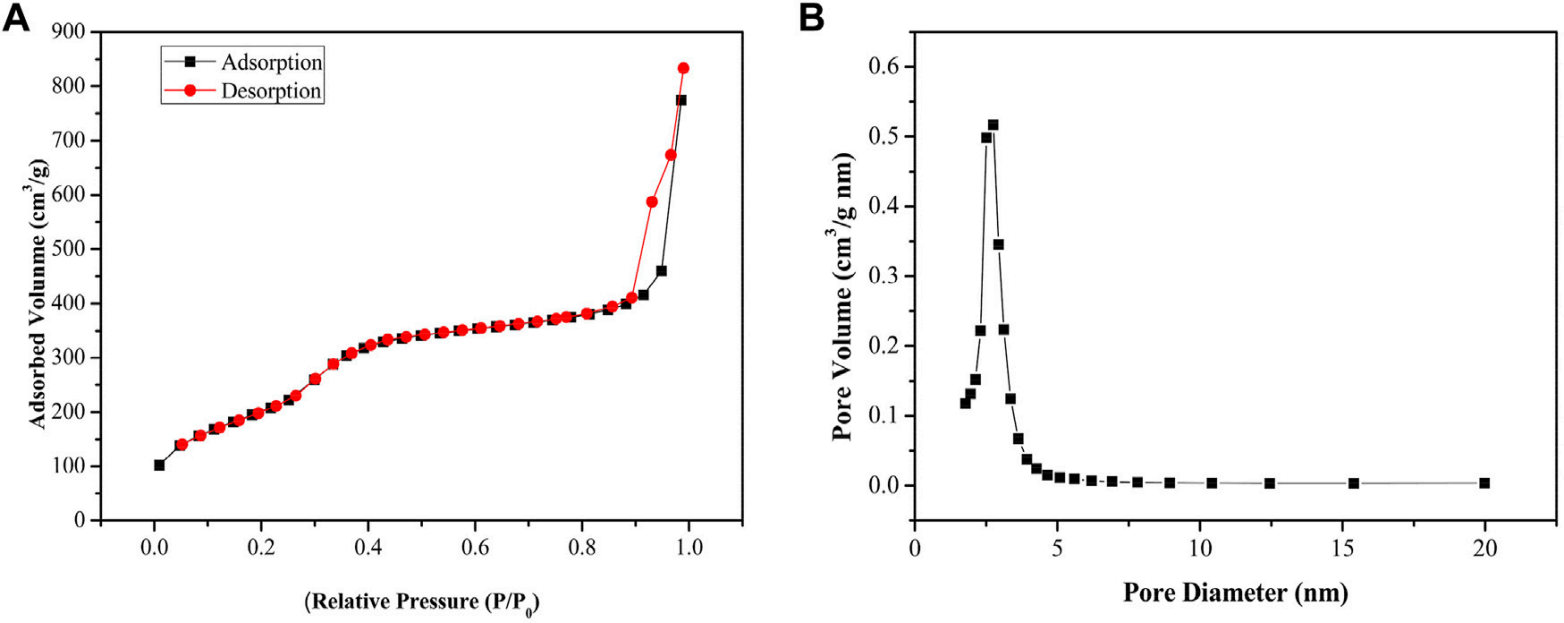
**(A)** Nitrogen adsorption–desorption isotherms. **(B)** Pore size distribution curves, indicating an average pore size of about 3.49 nm for these Gd_2_O_3_@mesoporous silica nanoparticles.

Both surface area and pore distribution are critical for evaluating mesoporous materials; thus, all of the N_2_ adsorption–desorption isotherms of Gd_2_O_3_@MSN using BJH (Figures 4A,B) were evaluated. The samples exhibit typical type IV curves with an evident hysteresis loop, confirming its mesoporous nature (López et al., 2006; Liu et al., 2015; Philippart et al., 2017; Francesca et al., 2018; Naseem et al., 2020; Zhou et al., 2020). The BET test results also suggest that the surface area of the MSN was around 822.96 m^2^/g, making them much larger than previous versions of similar compounds (Shao et al., 2011). The average pore size of these MSN was 3.49 nm and BJH revealed that they exhibited increased pore volume, 0.72 cm^3^g^−1^ compared to seminal MSN materials.

**FIGURE 5:**
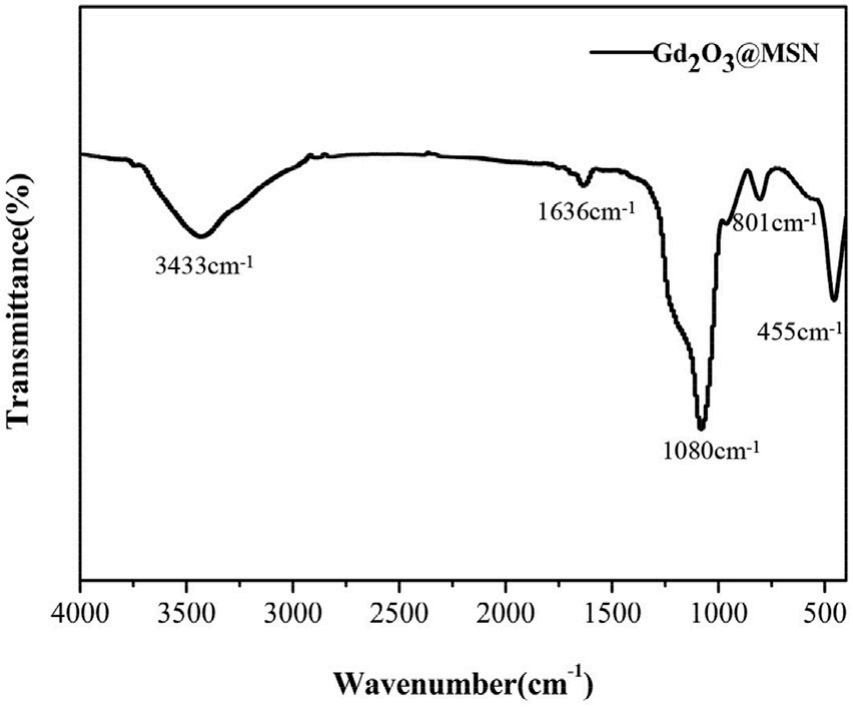
Fourier transform infrared refraction spectroscopy of Gd_2_O_3_@mesoporous silica nanoparticles.

These structures were then validated using an FT-IR spectrometer. The FT-IR spectra for these NPs produced peaks at 801 cm^−1^ and 455 cm^−1^ corresponding to Si-OH stretching, a peak at 1,080 cm^−1^ corresponding to Si-O-Si stretching vibration, and a peak at 3,433 cm^−1^ corresponding to the absorption bands of the hydroxyl group on MSN surface. These spectra also included a peak at 1,636 cm^−1^, corresponding to the bending vibration of -OH. Taken together, these patterns were representative of the characteristic absorption peaks of *MSN* materials (Figure 5) (Ni et al., 2016; He et al., 2019; Jiang et al., 2019; Cai et al., 2020).

### 3.2 T_1_ Relaxivity and Gd Stability

**FIGURE 6:**
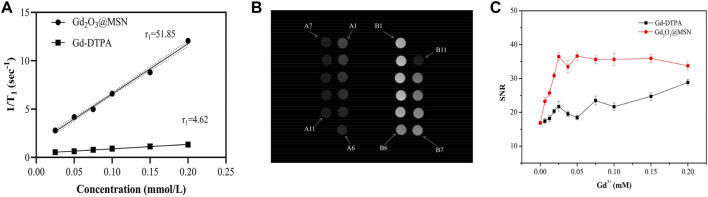
Magnetic resonance contrast enhancement analysis of Gd_2_O_3_@mesoporous silica nanoparticles (MSN) and Gd-gadolinium diethylene triamine pentaacetate (DTPA). Analysis of the relaxation rate *r*_*1*_ versus gadolinium ion concentration for Gd_2_O_3_@MSN and Gd-DTPA in a 0.5T magnetic field **(A)**; T_1_-weighted phantom images produced from Gd_2_O_3_@MSN and Gd-DTPA under a 3T field **(B)**; **(C)** signal-to-noise ratios of Gd_2_O_3_@MSN and Gd-DTPA at varying Gd concentrations.

The ability of pure Gd_2_O_3_@MSN was evaluated to enhance *T*_*1*_ contrast using a 0.5 T NMI20 Analyst NMR system. The mean *r*_*1*_ value (0.5 T) for Gd_2_O_3_@MSN was 51.85 ± 1.38 mM^−1^s^−1^, which was much larger than that of Gd-DTPA (4.62 ± 0.43 mM^−1^s^−1^) (Figure 6A). The geometrical confinement of these structures resulted in an approximately 12-fold increase in the Gd_2_O_3_@MSN *r*_*1*_ value when compared to that of the commercial Gd-DTPA, which suggested that these MSN could potentially be used as a *T*_*1*_ contrast agent. The stronger contrast effect may be attributed to the tuneable pore structure which facilitates ready access of the water molecules (Parvesh et al., 2012), allowing for multiple water molecules to be coordinated with each Gd centre, decreasing Gd rotation within the framework (Lin et al., 2004; Jiang et al., 2019).

Figure 6B displayed the *in-vitro T*_*1*_-weighted images obtained using Gd_2_O_3_@MSN samples (numbered B1–B11) produced using different Gd concentrations, with these images clearly demonstrating that increasing Gd^3+^ concentration facilitates increased image brightness. Figure 5B describes similar *in-vitro T*_*1*_-weighted images using Gd-DTPA (numbered A1–A11) at the same concentrations (per Gd atom) as the Gd_2_O_3_@MSN images. This data clearly shows a significant increase in brightness in the Gd_2_O_3_@MSN images compared to the Gd-DTPA (Supplementary Table S1) images at the same concentration when captured at an MRI intensity of 1972.36 ± 5.57 a. u. (Supplementary Table S2). Next, the signal-to-noise ratio (SNR) was calculated to evaluate the contrast enhancement using finely tuned and broadly representative ROIs within the transverse images. The SNR for each image was then evaluated using the single image method described by the American Association of Physicists in Medicine (AAPM), using the following equation: SNR= (S_central_–S_background_)/SD (AAPM Quality, 1990). These evaluations revealed that the SNR value of the Gd_2_O_3_@MSN images was much higher than that of Gd-DTPA images (Figure 6C).

Given the documented toxicity of free Gd^3+^ ions, its stability was evaluated within these MSN constructs as the first step for estimating its likely toxicity (Edyta et al., 2019; Lubinda et al., 2018). This was completed by placing Gd_2_O_3_@MSN (Gd^3+^, 100 mg/L) into PBS at different pH values (7.4, 5.5, and 4.5) and incubating these solutions for 48 h at 37°C, with both the pH and temperature designed to emulate various physiological conditions, including normal blood (pH 7.4), endosomes (pH 5.5) and lysosomes (pH 4.5) (Qi et al., 2019; Zhao et al., 2019). ICP-MS was then used to detect any free Gd ions in these solutions (Supplementary Figure S1). These evaluations revealed that there were very few free Gd^3+^ ions in any of these solutions (<1%) suggesting that Gd_2_O_3_@MSN was stable in these simulated *in-vitro* environments.

### 3.3 *In-Vitro* Cytotoxicity Studies

**FIGURE 7:**
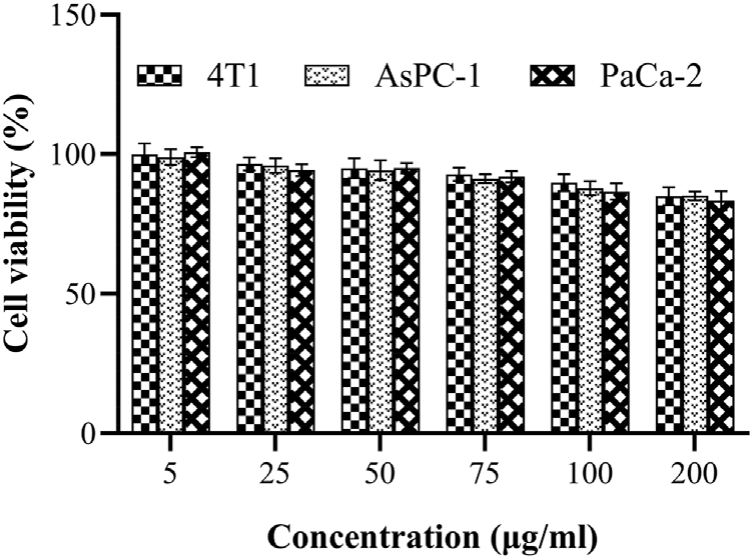
Cell viability of AsPC-1, PaCa-2 and 4T1 cells as determined by CCK-8 assay after treatment with Gd_2_O_3_@mesoporous silica nanoparticles (5, 25, 50, 100, 150 and 200 μg/ml).

The cytotoxicity of Gd_2_O_3_@MSN was estimated using an *in-vitro* assay of AsPC-1, PaCa-2 and 4T1 cells (Figure 7). CCK-8 assay revealed that there was no significant cytotoxicity following 24 h of exposure to any of the NPs within the described concentration range, suggesting that these NPs exhibit little toxicity toward AsPC-1, PaCa-2 and 4T1 cells (*p* > 0.05).

### 3.4 *In-Vivo* Safety Evaluation

**FIGURE 8:**
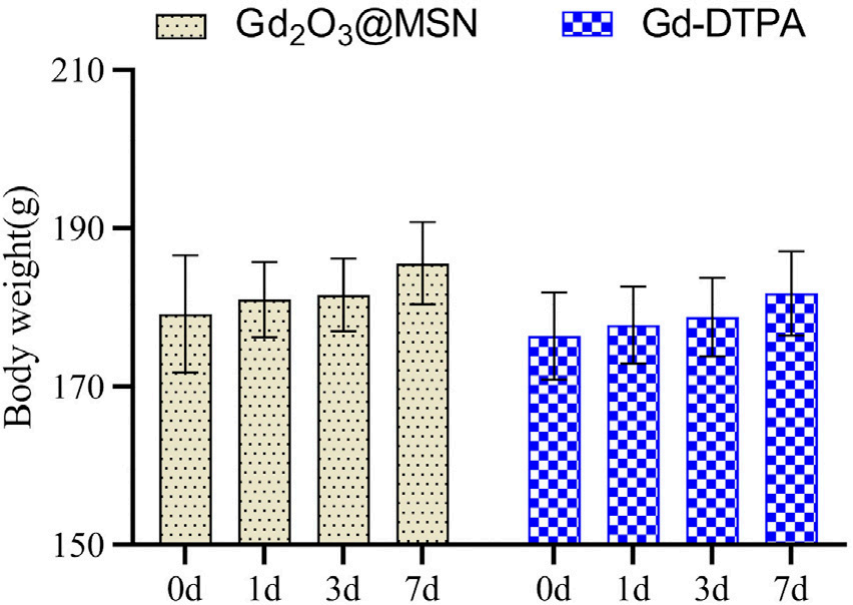
Weight changes in rats after 1 week of exposure to Gd_2_O_3_@mesoporous silica nanoparticles. Data are expressed as the mean ± standard deviation (*n* = 5).

**FIGURE 9:**
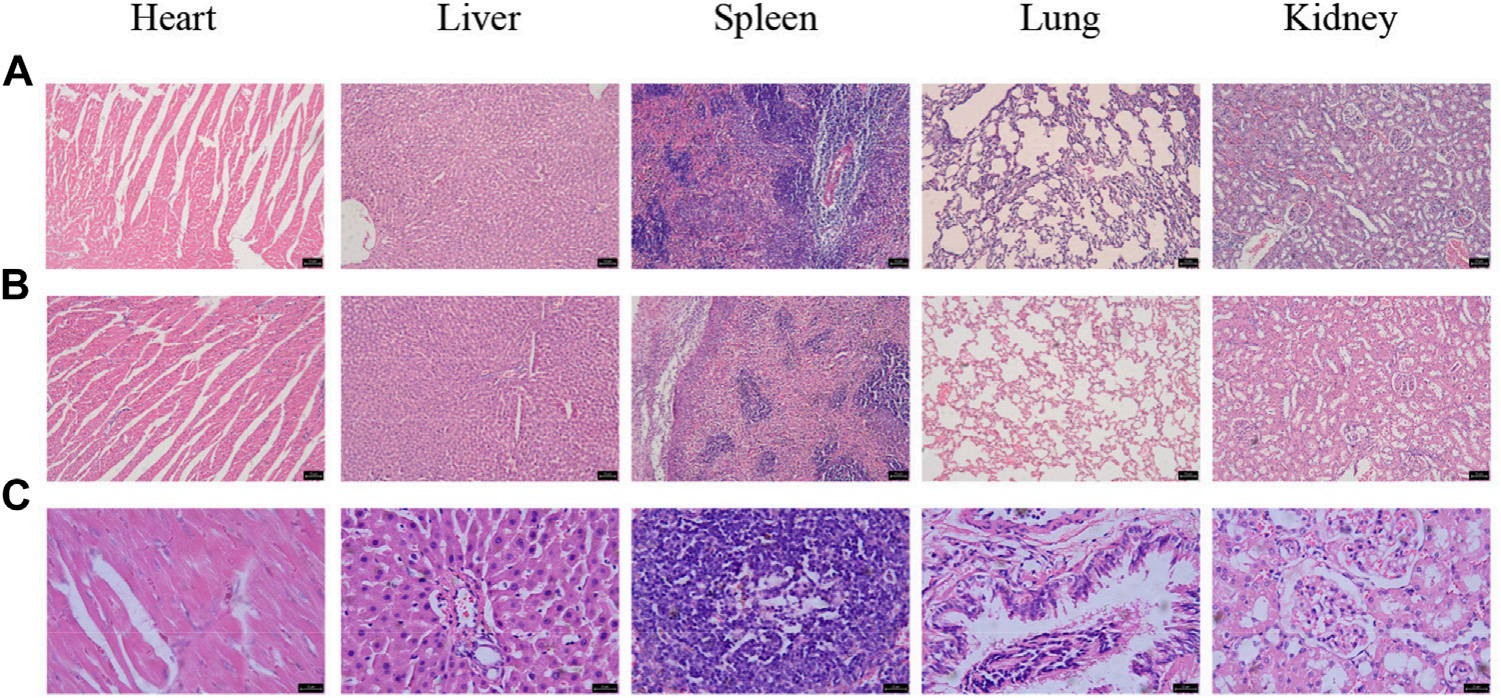
Haematoxylin and eosin staining of the heart, liver, spleen, lung and kidneys from **(A)** Gd- gadolinium diethylene triamine pentaacetate treated Sprague–Dawley rats, scale bar: 75 μm; **(B,C)** Gd_2_O_3_@MSN treated SD rats, scale bar: 75, 25 μm (7 days post-injection).

Healthy SD rats were treated with a high dose (100 mg/kg) of Gd_2_O_3_@MSN solution via intravenous (i.v.) injection and then monitored for up to 1 week to assess the *in-vivo* toxicity of Gd_2_O_3_@MSN. Negligible systemic toxicity or side effects were observed when using bodyweight measurement as a comparator (Figure 8) for any of these treatments with all the rats surviving the full-time course. Next, the main organs (heart, liver, spleen, lung, kidney) were harvested from these animals 7 days post-injection and were used to complete the histological assessment of these tissues using haematoxylin and eosin (H&E) staining. H&E evaluations revealed no clear changes in any of these tissues indicating acute (7 days) toxicity in response to Gd_2_O_3_@MSN exposure (Figure 9). A mini blood panel was also used to evaluate the potential cytotoxic effects of these NPs with all of the data suggesting the general health of these animals. (Table 2). These biochemical parameters included glutamic aspartate transaminase (AST), alanine aminotransferase (ALT), total bilirubin (T-BIL), blood urea nitrogen (BUN) and creatinine (Cr), all of which had no significant differences when compared with the control group (Gd-DTPA) on day 7 post-injection; thus, confirming that the primary function of both the kidneys and liver was not impaired post Gd_2_O_3_@MSN treatment. These results confirm the earlier findings around toxicity and support our hypothesis that this NP is well tolerated in living systems (Bong and Jaeyun, 2019; Yuan et al., 2020).

**TABLE 2:** Blood chemistry results for Sprague–Dawley rats injected with Gd2O3@mesoporous silica nanoparticles (*n* = 5).

	AST (U/L)	ALT (U/L)	T- BIL (μmol/L)	BUN (mmol/L)	Cr (μmol/L)
Gd_2_O_3_@MSN	151.08 ± 22.88	125.61 ± 17.46	0.68 ± 0.13	8.86 ± 1.65	31.32 ± 4.17
Gd-DTPA	142.65 ± 17.52	118.63 ± 16.42	0.72 ± 0.13	9.41 ± 2.01	29.42 ± 3.92

### 3.5 *In Vivo* Magnetic Resonance Imaging Studies

**FIGURE 10:**
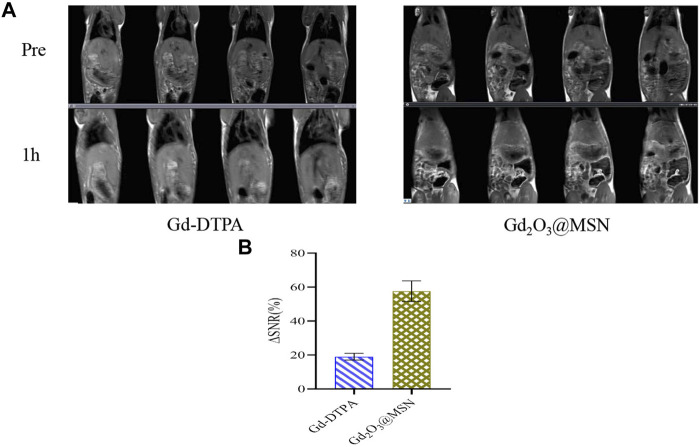
**(A)** *T*_*1*_-weighted *in-vivo* magnetic resonance imaging of rats (coronal plane) collected before and 1 h after intravenous injection with Gd-gadolinium diethylene triamine pentaacetate (DTPA) or Gd_2_O_3_@mesoporous silica nanoparticles (MSN) (at a dose of 0.5 mg Gd per kg of body weight); **(B)** Signal-to-noise ratios 1 h after intravenous injection of Gd-DTPA or Gd_2_O_3_@MSN.

As-prepared contrast materials were injected into healthy SD rats for *in-vivo* imaging to evaluate Gd_2_O_3_@MSN as an *in-vivo* MRI contrast agent. Previous studies have shown that MSN adsorbs plasma proteins and interacts strongly with the tissue-resident macrophages in the mononuclear phagocyte system (MPS), leading to rapid blood clearance and accumulation in the liver and spleen (Gao et al., 2011; Ekkapongpisit et al., 2012; Li et al., 2016; Wang and Sun, 2020). Given this, we used the likely accumulation of our NPs in the hepatic Kupffer cells to determine our ROIs, focusing on the liver region. *T*_*1*_-weighted images were collected before and 1 h after injection using a 3.0 T clinical MRI scanner. Our evaluations revealed that the liver region exhibited a significantly increased signal in animals treated with Gd-DTPA and Gd_2_O_3_@MSN (Figure 10A) when compared to the control. However, a comparison of the Gd-DTPA and Gd_2_O_3_@MSN images revealed a distinct increase in the liver signal from the novel contrast agent group, likely as a result of its higher *r*_*1*_ value and increased accumulation in the liver due to its larger size. Next, the signal intensity (SI) was calculated to evaluate the contrast enhancement via the comprehensive evaluation of the ROIs in each of the transverse images (Figure 10B). The ΔSNR value for Gd_2_O_3_@MSN was (57.54 ± 6.10)%, which was much higher than that of Gd-DTPA (18.98 ± 1.96)%, confirming the significant improvement in MRI *T*_*1*_ contrast when using Gd_2_O_3_@MSN for evaluating the liver.

## 4 Conclusion

To summarize, we successfully designed and fabricated a novel Gd_2_O_3_@MSN contrast reagent by one-step synthesis that produced a significant improvement in *T*_*1*_ contrast capacity primarily mediated by the increased geometrical confinement effect of this product. The one-step synthesis method is easy and cost-effective, which would be beneficial for the development of novel MRI contrast agents and for expanding the potential use of high-performance contrast agents for MRI and diagnosis.

## Data Availability

The original contributions presented in the study are included in the article/Supplementary Material, further inquiries can be directed to the corresponding authors.

## Fundings

This research was funded by Medical Sciences Project of Qiqihar Academy, grant number QMSI2019L-03, Basic scientific research business expenses of Heilongjiang Provincial Department of Education, grant number 2019-KYYWF-1251.
