# Supplementary material for: Gadolinium-Coated Mesoporous Silica Nanoparticle for Magnetic Resonance Imaging
Source: Front Chem. 2022 Feb 15;10:837032. doi: 10.3389/fchem.2022.837032 (PMC8885602; doi:10.3389/fchem.2022.837032)
Supplement: Supplementary file 1 [file DataSheet1.doc]

***Supporting information for***

**Gadolinium-coated mesoporous silica nanoparticle for magnetic resonance imaging**

**Zhongtao Li^1^,Jing Guo^1^,Mengmeng Zhang^1^, Guohua Li^2^*and Liguo Hao^1 3^***

1. Department of Molecular Imaging, School of Medical Technology, Qiqihar Medical University, Qiqihar, Heilongjiang, 161006, China

2.Department of Radiology, The First Affiliated Hospital of Qiqihar Medical University, Qiqihar, Heilongjiang, 161041, China

3.Department of Molecular Imaging, The First Affiliated Hospital of Qiqihar Medical University, Qiqihar, Heilongjiang, 161041, China

*Corresponding author (GH L): E-mail: liguohua@qmu.edu.cn

*Corresponding author (LG H): E-mail: haoliguo@qmu.edu.cn

Table of contents

Page

Figure S1 S2

Figure S2 S3

Figure S3 S4

Table S1 S4

Table S2 S6

Table S3 S7


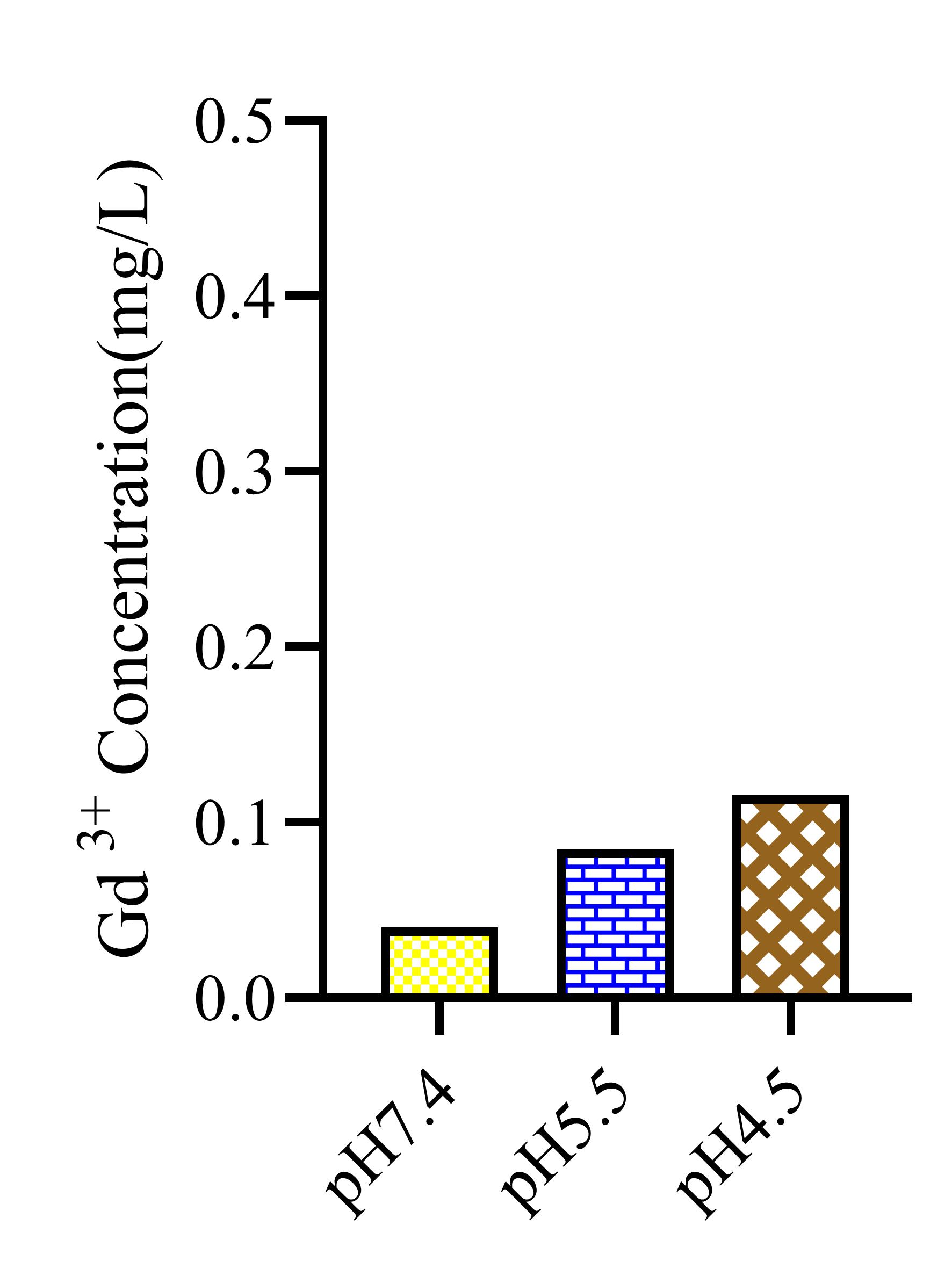


**Figure S1.** Release of Gd^3+^ in PBS buffers at various pH value(pH 7.4, pH 5.5, pH 4.5)


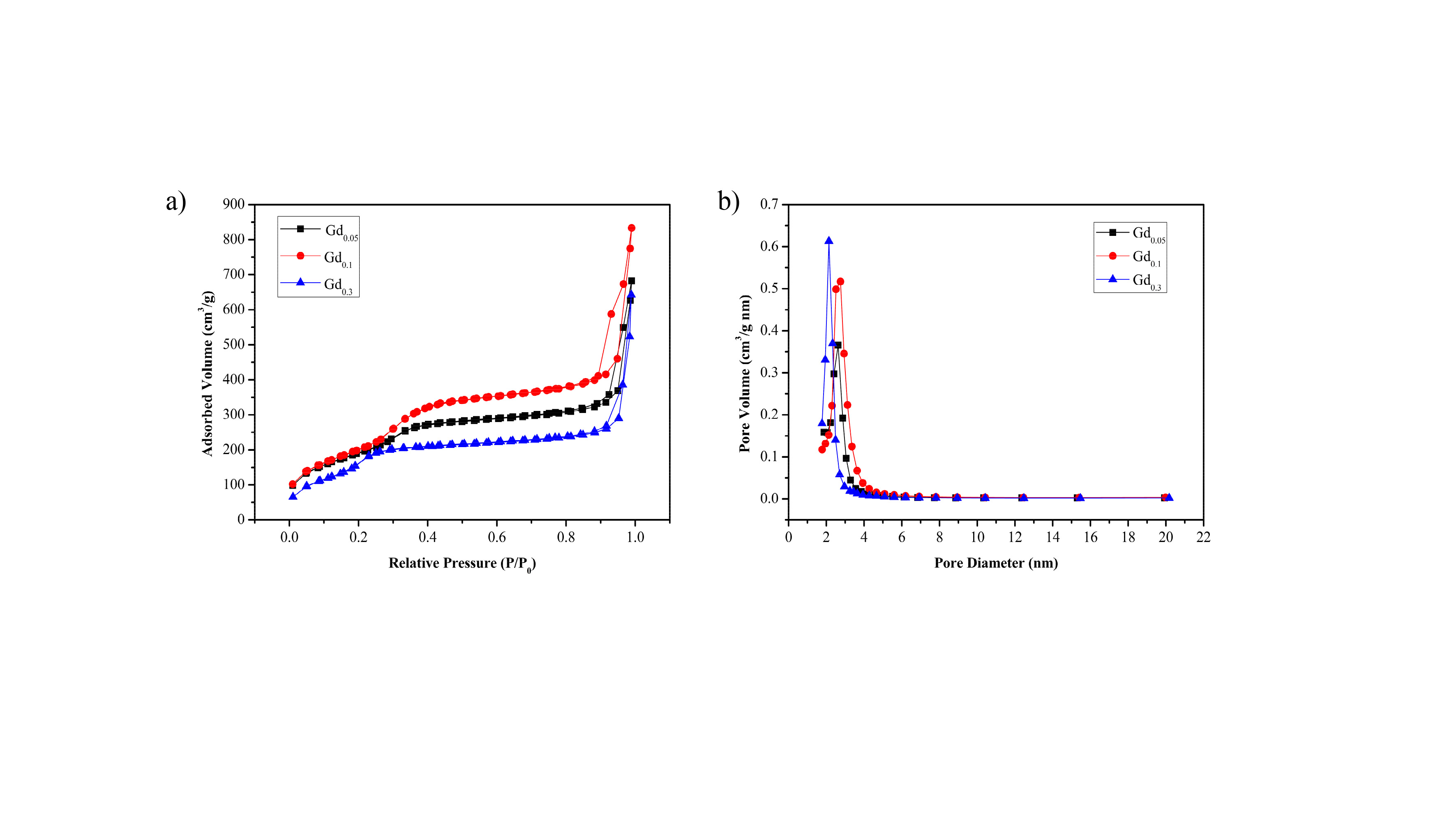


**Figure S2.** Properties analysis of Gd_2_O_3_@mesoporous silica nanoparticles (Gd_0.05_, Gd_0.1_ and Gd_0.3_ are respectively doped with GdCl_3_·6H_2_O 50mg, 100mg and 300mg ).





**Figure S3.** X-ray diffraction of Gd_2_O_3_@mesoporous silica nanoparticles

**Table S1**. MRI intensity for varying concentrations of commercial Gd-DTPA

| Sample no. | MRI intensity (a.u.) |
| --- | --- |
| Gd1(0.2) | 873.19±1.76 |
| Gd2(0.15) | 771.21±2.43 |
| Gd3(0.1) | 694.30±5.03 |
| Gd4(0.075) | 653.07±2.63 |
| Gd5(0.05) | 585.27±2.27 |
| Gd6(0.0375) | 577.31±1.46 |
| Gd7(0.025) | 508.59±2.10 |
| Gd8(0.01875) | 493.93±1.44 |
| Gd9(0.0125) | 480.23±0.75 |
| Gd10(0.00625) | 473.26±1.04 |
| Gd11(0) | 473.53±1.12 |

Gd-DTPA (per Gd atom; mmol/l; a.u.= arbitrary units)

**Table S2.** MRI intensity for varying concentrations of Gd_2_O_3_@MSN

| Sample no. | MRI intensity (a.u.) |
| --- | --- |
| MSN1(0.2) | 1972.36±5.57 |
| MSN2(0.15) | 1965.98±5.67 |
| MSN3(0.1) | 1932.10±6.84 |
| MSN4(0.075) | 1865.47±5.53 |
| MSN5(0.05) | 1824.35±7.81 |
| MSN6(0.0375) | 1528.52±1.56 |
| MSN7(0.025) | 1504.81±8.75 |
| MSN8(0.01875) | 146682±1.68 |
| MSN9(0.0125) | 1385.57±3.71 |
| MSN10(0.00625) | 1324.11±4.86 |
| MSN11(0) | 480.41±3.43 |

Gd_2_O_3_@MSN (per Gd atom; mmol/l; a.u.= arbitrary units)

**Table S3.** Properties analysis of mesoporous silica nanoparticles. (Gd_0.05_, Gd_0.1_ and Gd_0.3_ are respectively doped with GdCl_3_·6H_2_O 50mg, 100mg and 300mg ).

|  | Surface Area (m^2^/g) | Pore Volume (cm^3^/g) | Pore Size (nm) |
| --- | --- | --- | --- |
| Gd_0.05_ | 723.53 | 0.57 | 3.14 |
| Gd_0.1_ | 822.95 | 0.72 | 3.49 |
| Gd_0.3_ | 738.88 | 0.45 | 2.43 |
